# Stem cell-derived extracellular vesicles: a potential intervention for Bronchopulmonary Dysplasia

**DOI:** 10.1038/s41390-024-03471-2

**Published:** 2024-09-09

**Authors:** Hala Saneh, Heather Wanczyk, Joanne Walker, Christine Finck

**Affiliations:** 1 Department of Neonatal Medicine, Connecticut Children’s Medical Center, Hartford, CT USA; 2 Department of Pediatrics, University of Connecticut Health Center, Farmington, CT USA; 3 Department of Pediatric Surgery, Connecticut Children’s Medical Center, Hartford, CT USA

## Abstract

**Abstract:**

Despite advances in neonatal care, the incidence of Bronchopulmonary Dysplasia (BPD) remains high among extreme preterm infants. The pathogenesis of BPD is multifactorial, with inflammation playing a central role. There is strong evidence that stem cell therapy reduces inflammatory changes and restores normal lung morphology in animal models of hyperoxia-induced lung injury. These therapeutic effects occur without significant engraftment of the stem cells in the host lung, suggesting more of a paracrine mechanism mediated by their secretome. In addition, there are multiple concerns with stem cell therapy which may be alleviated by administering only the effective vesicles instead of the cells themselves. Extracellular vesicles (EVs) are cell-derived components secreted by most eukaryotic cells. They can deliver their bioactive cargo (mRNAs, microRNAs, proteins, growth factors) to recipient cells, which makes them a potential therapeutic vehicle in many diseases, including BPD. The following review will highlight recent studies that investigate the effectiveness of EVs derived from stem cells in preventing or repairing injury in the preterm lung, and the potential mechanisms of action that have been proposed. Current limitations will also be discussed as well as suggestions for advancing the field and easing the transition towards clinical translation in evolving or established BPD.

**Impact:**

- Extracellular vesicles (EVs) derived from stem cells are a potential intervention for neonatal lung diseases. Their use might alleviate the safety concerns associated with stem cell therapy.
- This review highlights recent studies that investigate the effectiveness of stem cell-derived EVs in preclinical models of bronchopulmonary dysplasia. It adds to the existing literature by elaborating on the challenges associated with EV research. It also provides suggestions to advance the field and ease the transition towards clinical applications.
- Optimizing EV research could ultimately improve the quality of life of extreme preterm infants born at vulnerable stages of lung development.

## Introduction

Bronchopulmonary Dysplasia (BPD), also known as Chronic Lung Disease (CLD) of prematurity, is a multifactorial disease characterized by an inflammatory cascade that severely injures the preterm lung and arrests its normal development.^1^ Clinical implications such as impaired lung function can persist into adulthood.^2^ Multiple interventions, including protective ventilation strategies, caffeine, diuretics, and steroids, have been used to reduce the risk of BPD. However, the disease remains widely prevalent in preterm infants, affecting around 40% of those born before 28 weeks’ gestation.^3,4^

With the significant growth in the field of regenerative medicine in the past couple decades, multiple experimental pre-clinical studies have shown a promising role for stem cell therapy in reducing inflammatory changes and restoring morphogenesis following hyperoxia-induced lung injury.^5^ Recent reports have suggested that the benefits of stem cells may be largely mediated by paracrine mechanisms involving the secretion of extracellular vesicles (EVs) in the milieu. These EVs have been shown to contain biochemical components that promote tissue regeneration following injury.^6^ We aim to review the available literature supporting the use of stem cell-derived EVs as a potential intervention for BPD. We will also summarize some of the remaining challenges and provide recommendations for advancing the field.

### The evolving pathophysiology of Bronchopulmonary Dysplasia

**Fig. 1:**
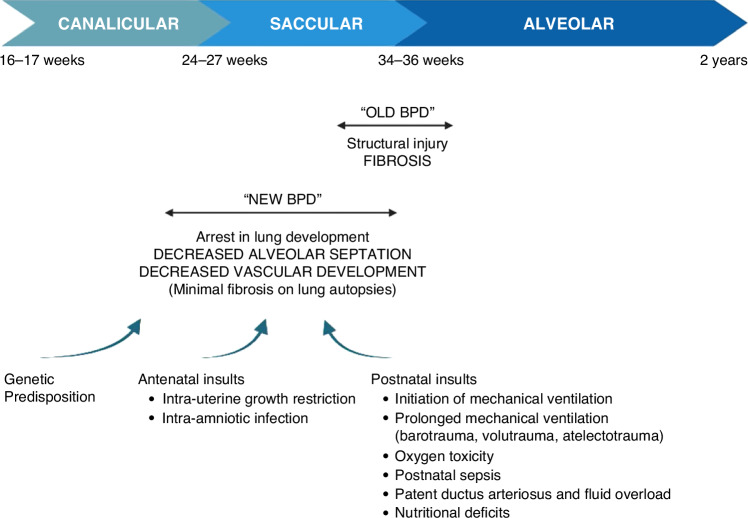
Stages of lung development and the evolving pathophysiology of BPD. With the increased survival of extreme preterm infants and the advances in neonatal care, the definition and pathophysiology of BPD have changed over time. Histologic characteristics of “old BPD” and “new BPD” are presented. Multiple intrinsic, antenatal, and postnatal risk factors contribute nowadays to the development of the disease [Created with BioRender].

BPD was initially described by Northway in 1967 at a time when the use of mechanical ventilation for preterm infants was still at its very beginning.^7^ This “old” BPD was defined as a severe lung injury induced by oxygen exposure and mechanical ventilation. Parenchymal fibrosis was a major finding on lung autopsies. With the improvement in neonatal care over the past decades, the survival of extreme preterm infants (born before 28 weeks’ gestation) has significantly increased, and the population affected by BPD has changed.^8^ Multiple factors other than oxygen exposure and mechanical ventilation are nowadays contributing to the disease.^9^ This “new” BPD is defined as an arrest in lung development and is characterized histologically by a decrease in alveolar septation and vascular development ^7,10,11^(Fig. 1).

Mechanical ventilation and oxygen supplementation are known risk factors for injury and inflammation in the developing lung. However, some extreme preterm infants develop BPD without previously requiring significant oxygen supplementation or mechanical ventilation, which highlights the evidence that other risk factors play a significant role in activating the inflammatory cascade.^12^ Multiple intrinsic factors (such as male sex and genetic predisposition), pre-natal exposures (such as intrauterine fetal growth restriction and intra-amniotic infection), and post-natal insults (such as sepsis, patent ductus arteriosus, mechanical ventilation, and oxygen exposure) affecting preterm infants at an early stage of their lung development can cause an arrest in both alveolarization and vascularization and lead to chronic lung disease ^13–15^(Fig. 1).

New BPD is not a single disease. Multiple phenotypes have been identified. While enlarged airspaces and decreased number of alveoli were classically described as pathognomonic findings,^7^ parenchymal disease is only one component of BPD. Airway narrowing and vascular remodeling are two other components that also play an important pathophysiologic and clinical role in disease development and progression. Using a phenotypic approach to characterize BPD at a specific point of time, based on the presence or absence of parenchymal, airway and vascular involvement, is thus crucial to determine the most appropriate management at that time. The term “evolving BPD” refers here to the ongoing lung disease in preterm infants who have not yet reached 36 weeks of corrected postmenstrual age to fulfill the definition criteria of “established BPD”.^16^ Many affected infants end up having overlapping phenotypic features, with up to 32% of patients with established disease having all three components combined.^10^ Based on this information, therapeutic interventions capable of restoring normal parenchymal, airway and vascular development are warranted. For a treatment modality to be successful, it needs to have regenerative properties that can be targeted towards all three components.

## Stem cell therapy for Bronchopulmonary Dysplasia

### Exogenous stem cells for the treatment of *Bronchopulmonary Dysplasia*: from preclinical data to clinical trials

Preclinical studies reveal promising results in the amelioration of hyperoxia-induced lung injury following exogenous stem cell administration to animal models.^17,18^ Various types of stem cells have been investigated, including amnion epithelial cells (AECs), mesenchymal stromal cells (MSCs), and human-induced pluripotent stem cells (hiPSCs). A reduction in inflammatory changes and a partial restoration of lung architecture following hyperoxia-induced injury were noted with all treatments.^19–21^ Survival and exercise capacity were also improved over time.^22^ Stem cell-based BPD research is however impacted by the significant heterogeneity existing among the available studies in terms of animal model, concentration and duration of oxygen exposure, stem cell source, dosage and route of administration, and methods to assess efficacy.^5^ Preclinical studies need to be standardized to obtain reproducible findings and increase the chances for clinical translation. Nonetheless, a few clinical trials were conducted or proposed in the past few years to study the efficacy and safety of stem cell therapy in the prevention and/or treatment of lung injury in preterm infants.^23^

In a US-based phase I dose-escalation trial published in 2019, 12 extremely low birth weight infants received a single intra-tracheal dose of human umbilical cord-derived MSCs at 5–14 days of life; the treatment appeared to be safe and well-tolerated. Among these infants, 10 were diagnosed with severe BPD at 36 weeks of corrected gestational age. However, since the trial was designed as a safety study, there was no control group, and thus no reliable conclusion could be made regarding the efficacy of MSCs in attenuating lung injury.^24^

A Korean phase II clinical trial published in 2021 randomized 60 mechanically ventilated extreme-preterm infants at 5–14 days of life, to receive either an intratracheal treatment of allogeneic umbilical cord-derived MSCs or normal saline. Disease severity was reduced in the treatment group born between 23- and 24-weeks’ gestation, but not in infants born at a more advanced gestational age. Patients were monitored for 6 months following the treatment, and no serious adverse events were reported.^25^ This study is however limited by the small sample size and the less-than-expected rate of severe BPD in the enrolled infants. Also, although the difference in sex distribution among the groups did not reach statistical significance, the control group had more male infants enrolled than the treatment group. Male sex is known to be associated with an increased severity of preterm lung disease.

Additional phase I and II clinical trials are ongoing in several countries. The currently active trials registered in the National Institute of Health database are listed in Table 1.

**Table 1:** Ongoing clinical trials investigating stem cell use in preterm lung disease.

Clinical trial	Phase	Location	Stem cell source	Status
NCT04255147	Phase I	Canada	Allogeneic umbilical cord-derived MSCs	Recruiting
NCT03392467	Phase II	Korea	Allogeneic umbilical cord-derived MSCs (*PNEUMOSTEM*)	Recruiting
NCT04003857	Phase II	Korea	Allogeneic umbilical cord-derived MSCs (*PNEUMOSTEM*)	Recruiting
NCT03645525	Phases I and II	China	Allogeneic umbilical cord-derived MSCs	Recruiting
NCT04062136	Phase I	Taiwan	Allogeneic umbilical cord-derived MSCs (*UMC119-01*)	Recruiting
NCT02443961	Phase I	Spain	MSCs (unspecified tissue source, unspecified whether autologous or allogeneic)	Completed, no results posted yet

Only those registered in the NIH database and active are listed.

### Safety concerns associated with exogenous stem cell therapy

Despite the promising results of the abovementioned studies, serious concerns need to be considered when building the framework for clinical translation, including immunogenicity, tumorigenicity, and phenotypical divergence under inflammatory environmental conditions.

Immunogenicity is known to be a major problem with any allogeneic stem cell source. Despite the low level of expression of the Major Histocompatibility Complex class 1 (MHC-1) on the surface of embryonic stem cells (ESCs), it still renders them susceptible to lysis by host Natural Killer (NK) cells. Allopeptides derived from MHC antigens can also be released and presented on the surface of Antigen Presenting Cells, triggering a T-cell response.^26^ Theoretically, immunogenicity should be prevented by using autologous stem cell sources, but this might not be the case. Reports have shown that autologous MSCs administered after a period of in vitro culture can also be targeted by activated NK cells, while resident MSCs are not. A possible explanation is that autologous MSCs may have undergone phenotypic changes during their in vitro culture, rendering them vulnerable to the patient’s own immune system.^27^

Tumorigenicity is another potential concern, mostly reported with undifferentiated pluripotent stem cells. In an experimental murine model, teratoma formation was noted within a 10-week period following the subcutaneous injection of hiPSCs.^28^ This potential risk depends on multiple factors, including the administered amount of undifferentiated hiPSCs and the cell line used, and is largely attributed to epigenetic memory and potential genomic instability.^28–30^

Additionally, the function of progenitor/stem cells is highly regulated by complex micro-environmental stimuli. It has been shown that the endogenous pool of lung-derived MSCs may, under inflammatory conditions, divert into a myo-fibroblastic phenotype, and promote more damage than repair.^31^ What if exogenous stem cells administered in a milieu already affected by inflammation also transform into a detrimental phenotype? In this case, it may be safer to use the proposed treatment as a preventive measure at the time of birth before the inflammatory cascade is triggered, rather than later. But would it still be effective if used preventively? Most studies conducted so far have focused on administering progenitor/stem cells during or following hyperoxia exposure. Administration in the early inflammatory phase seems to offer better protection against chronic lung injury than treatment in progressive stages of the disease.^23,32^ Therefore, there may be significant merit in the early administration of stem cells prior to the occurrence of any additional postnatal lung injury.

### The rationale behind the use of acellular therapeutic approaches for treating Bronchopulmonary Dysplasia

The concerns associated with stem cell therapy might hinder its clinical translation in preterm infants. The past decade has seen the emergence of cell-free approaches as a potential alternative regenerative treatment for several conditions, such as cancer, aging, and autoimmune diseases.^33–35^ Could these acellular therapies also be applied to Bronchopulmonary Dysplasia?

When immunofluorescent staining was used to track umbilical cord-derived MSCs after their intra-tracheal administration into rat pups following hyperoxic exposure, low levels of engraftment were noted in recipient lungs. Levels became almost undetectable within four days of the intervention.^6^ Regardless, stem cells were effective in attenuating lung injury, which suggests that their therapeutic benefits are more likely to be mediated by their secretome. Paracrine mechanisms have been further investigated by using cell-free MSC-conditioned media to treat animal BPD models. Interestingly, similar promising results were observed in terms of tissue regeneration,^6,36^ favoring the hypothesis that MSCs act primarily via paracrine mechanisms by releasing factors or particles capable of tissue repair.

Identifying these particles and using them to prevent or treat BPD might mitigate some of the concerns associated with the use of stem cells, while retaining their effectiveness in tissue repair. Theoretically, Major Histocompatibility Complexes (MHC) are less abundant in a cell secretome than on the surface of the cell itself. Thus, acellular approaches are less likely to be immunogenic.^37–39^ Tumorigenicity is unlikely given that cell-secreted particles or vesicles cannot self-replicate. Divergence of the cell towards a detrimental phenotype is also no longer an issue with acellular approaches.

## Extracellular Vesicles (EVs): Bioactive material in nano-packages

**Fig. 2:**
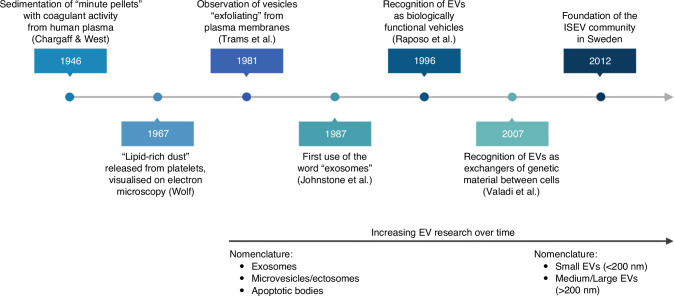
Major historical timepoints in EV research. Key points in the history of EV research are illustrated in this timeline [Created with BioRender].

Extracellular vesicles, or EVs, are cell-derived nanoparticles, secreted by most eukaryotic cells. They were first described in 1946 by Chargaff and West as “minute pellets” obtained following high-speed centrifugation of human plasma.^40^ The major historical timepoints in EV discovery are illustrated in Fig. 2.^40–47^ Johnstone et al. used the word “exosomes” for the first time in 1987 to refer to particles released from reticulocytes by an externalization mechanism.^43^ Further studies showed that “exosomes” are only a subtype of EVs; they are produced via the endosomal pathway, then secreted following the fusion of multivesicular bodies with the plasma membrane. Other subtypes include “microvesicles or ectosomes” (formed by direct blebbing of plasma membranes) and “apoptotic bodies” (resulting from cellular death) (Table 2).^33,35,48,49^

**Table 2:** Traditional classification of EVs based on their biogenesis pathways.

	“Exosomes”	“Ectosomes/microvesicles”	“Apoptotic bodies”
Size	30 to 150 nanometers	100 to 1000 nanometers	More than 1000 nanometers
Biogenesis	Fusion of multivesicular bodies with the plasma membrane	Outward blebbing of the plasma membrane	Release during cellular shrinkage and death
Content	Lipids, proteins, mRNA, miRNA	Lipids, proteins, mRNA, miRNA	Organelles, nuclear fractions, lipids, proteins, DNA, RNA
Surface markers	Tetraspanins CD9, CD63 and CD81 and Flotillin-1 are commonly reported to be present on the surface of “exosomes”, while ADP-ribosylation Factor ARF6 is more frequently linked to “ectosomes or microvesicles”. However, since studies have used different isolation techniques to separate EVs before characterizing them, ISEV continues to refrain from associating any specific marker with a distinct EV subtype.^51^		

Significant overlaps exist between categories,

*ADP* adenosine diphosphate, *ARF6* ADP-ribosylation factor 6, *CD* cluster of differentiation, *DNA* deoxyribonucleic acid, *EV* extracellular vesicle, *ISEV* international society for extracellular vesicles, *RNA* ribonucleic acid (*mRNA* messenger RNA, miRNA: microRNA).

Initially thought to be vehicles that eliminate waste and cytotoxic substances from cells, subsequent studies reported a pivotal role for EVs in cellular communication and cell signaling.^33,44^ Raposo et al. demonstrated that EVs act as transport vehicles for MHC II peptides, and are biologically functional in maintaining T cell memory.^46^ Further research showed that EVs not only carry proteins, but also nucleic acids, proposing a new mechanism of genetic exchange between cells via their secreted vesicles.^47^

The rapid growth in EV research led to the establishment in 2012 of the International Society of Extracellular Vesicles (ISEV), with the release of frequently updated guidelines to support and standardize EV research worldwide (Minimal Information for Studies of Extracellular Vesicles MISEV 2014^50^ and 2018^51^) While the term “exosomes” remains widely used in the literature to refer to EVs, ISEV continues to label them as “extracellular vesicles” given the lack of consensus on unique characteristics of different EV subtypes.^51^

Size and surface markers significantly overlap among EVs (Table 2), making it difficult to isolate specific subtypes without contaminating the sample. Although the cellular process leading to the formation of “exosomes” and “ectosomes” are different, both subtypes carry bioactive intraluminal cargo, including proteins (structural proteins, heat-shock proteins, enzymes) and nucleic acids (mRNAs, small interfering RNAs, long noncoding RNAs).^49^ Various isolation techniques are used worldwide, yielding different EV populations, and resulting in significant heterogeneity among studies investigating the same disease process.

While certain EV populations may be specifically effective in certain disease processes, other populations may be needed for other conditions.^52^ Therefore, generalization of findings should be done with caution. When studying the functionality of an EV population in a specific disease, it is important to characterize the sample in terms of quantity or abundance of vesicles, size distribution, protein composition, single vesicle analysis, non-vesicular co-isolated particles, and localization of the bioactive components (surface-bound or cytoplasmic),^51^ as each of these factors can influence the overall potential therapeutic benefit of EVs.

## Stem cell-derived EVs for neonatal lung injury: promises

In recent years, EVs have been widely investigated for both diagnostic and therapeutic applications. They are now recognized as important vehicles for intercellular communication and signaling. When released in certain pathological conditions, EVs can serve as potential diagnostic and prognostic biomarkers.^34^ EVs also contribute to vital physiological functions, including homeostasis, immune regulation, and tissue regeneration.^53^

Given the aforementioned concerns with stem cell therapy, the field of regenerative medicine has also become more attracted to EV research. Proteomic and transcriptomic profiling of MSC-derived EVs revealed the presence of proteins and miRNAs capable of suppressing inflammation by inhibiting multiple pro-inflammatory pathways,^54^ such as nuclear transcription factor-κB (NF-κB),^55^ toll-like receptor 4 (TLR4),^56^ and signal transducer and activator of transcription 3 (STAT3) ^57^signaling pathways. Additionally, MSC-EVs were found to promote macrophage polymerization into the M2 anti-inflammatory phenotype,^56^ making them a potential candidate for treating inflammatory diseases. EVs derived from hiPSCs were also found to carry proteins implicated in pathways that drive parenchymal and vascular lung development under both normal and pathological circumstances, such as hypoxia-inducible factor 1 alpha (HIF1α) activation and vascular endothelial growth factor (VEGF) signaling pathways.^58^ Stem cell-derived EVs are thus emerging as a potential intervention to regenerate lung tissue and attenuate the inflammatory process. Several recent studies have investigated the effectiveness of stem cell-derived EVs in repairing neonatal lung injury (Table 3).

**Table 3:** Studies investigating the effectiveness of stem cell-derived EVs in repairing neonatal lung injury.

Authors, Year	Disease model	EV source	EV isolation	EV characte-rization	EV dose used	Route timing of treatment	Outcomes
Willis et al., ^59^	Newborn term mice exposed to 75% O_2_ for 7 days	Human MSCs (umbilical cord, bone marrow)	dUC, TFF, DGC	NTA, TEM, WB	Cell equivalent: 0.5 ×10^6^ MSCs cultured over 36 h. NTA corresponding dose: 7.2-8.5 ×10^8^ particles.	Intravenous once at PND 4	-Decreased MLI. -Decreased fibrosis measured by septal collagen deposition. -Improved lung function. -Improved pulmonary hypertension. -Immunomodulation of lung macrophage phenotype.
Ahn et al., ^61^	Newborn term rats exposed to 90% O_2_ for 14 days	Human MSCs (umbilical cord)	dUC	SEM, TEM, NTA, WB	Protein amount: 20 μg of protein per dose of EV.	Intra-tracheal once at PND 5	-Improved alveolarization and angiogenesis. -Decreased apoptosis. -Decreased levels of proinflammatory cytokines (IL-1, IL-6, TNF-α). -Internalization of EVs into pericytes, macrophages, pneumocytes type 2. *These benefits were not seen with EVs derived from VEGF-knockdown MSCs, suggesting that EV effects are primarily mediated by VEGF transfer*.
Braun et al., ^62^	Newborn term rats exposed to 85% O_2_ for 14 days	Rat MSCs (bone marrow)	dUC	NTA, TEM, WB	Protein amount: 15 μg of protein per dose of EV. NTA corresponding dose: 3.4 ×10^9^ particles.	Intraperitoneal daily PND 1-15	-Decreased MLI. -Increased blood vessel number. -Inhibition of right ventricular hypertrophy (RVH). -Stimulation of capillary network formation in human umbilical vein endothelial cells in vitro. This effect was mediated by VEGF.
Chaubey et al., ^63^	Newborn term mice exposed to >95% O_2_ for 4 days	Human MSCs (umbilical cord of preterm donors born at 25-weeks/ 30-weeks’ gestation)	LSC, Filtration	TEM, NTA	Cell equivalent: 0.7 ×10^6^ MSCs cultured over 24 h. NTA corresponding dose: 4.5 ×10^8^ particles derived from 25-weeker MSCs, and 2.88 ×10^7^ particles derived from 30-weeker MSCs.	Intraperitoneal at PNDs 2 and 4	-Decrease in alveolar size, reduction in alveolar septal thickness (higher response with EVs derived from donors born at 25-weeks). -Decreased cell death. -Reduced blood vessel loss. -Decreased RVH. *TSG-6 (tumor necrosis factor alpha-stimulated gene-6) suggested as a potential mediator of the therapeutic effects of EVs*.
Porzionato et al., ^70,71^	Newborn term rats exposed to 60% O_2_ for 14 days	Human MSCs (umbilical cord) [culture in bioreactor]	TFF	NTA, FC, ELISA, T/B cell assays for activity testing	Cell equivalent: 2 ×10^6^ MSCs cultured over 36 h. NTA corresponding dose: 8 ×10^8^, 4.5 ×10^8^, 3 ×10^8^, 1.5 ×10^8^ particles per gram of body weight at PNDs 3, 7, 10, 21 respectively.	Intra-tracheal at PNDs 3, 7, 10, 21	-Increased total number of alveoli, -decreased mean alveolar volume. -Reduction in the medial thickness of small pulmonary vessels. -Anti-inflammatory macrophage (M2) polarization effect.
Mitchell et al., ^21^	Newborn term mice exposed to 75% O_2_ for 14 days	hiPSCs and diPSCs (alveolar-like cells)	Precipita-tion and immune-magnetic bead-based isolation	SEM, WB, FC	Flow cytometry: 7.5 ×10^4^ EV-bead complexes	Intra-orally once at PND15	No benefits of EV-bead complexes in reversing histologic damage resulting from hyperoxia exposure. Beads alone: detrimental effect.
Willis et al., ^93^	Newborn term mice exposed to 75% O_2_ for 14 days	Human MSCs (umbilical cord)	dUC, TFF, DGC	NTA, TEM (Immu-nogold labelling), WB	Cell equivalent: 0.5 ×10^6^ MSCs (early intervention group), 1 ×10^6^ MSCs (late intervention groups) cultured over 36 h. NTA corresponding dose: 6 ×10^8^ and 1.7 ×10^9^ particles respectively.	Intravenous •Single dose at PND 4 •Single dose at PND 18 •Weekly at PNDs 18, 25, 32, 39	With both early and late interventions: -Decreased MLI, -decreased fibrosis measured by septal collagen deposition. -Improved functional capacity. -Improvement of pulmonary vascular remodeling and decrease in right ventricular hypertrophy.
Li et al., ^89^	Newborn term rats exposed to 80% O_2_ for 14 days	Human MSCs (amnion-derived)	UC	TEM, WB	Protein amount: 300 ng of protein per dose of EV.	Intra-tracheal once at PND 7	-Decreased alveolar septal thickening. No significant improvement in MLI. -Attenuation of pulmonary edema. Reduction of inflammatory markers (IL-6, TNF-α) in bronchoalveolar fluid *These benefits were less pronounced than those obtained with whole MSCs*.
You et al., ^64^	Newborn term rats exposed to 85% O_2_ for 14 days	Human MSCs (umbilical cord)	UC, Filtration	NTA, TEM, WB	Protein amount: 20 μg of protein per dose of EV.	Intra-tracheal once at PND 7	-Decreased MLI. -Attenuation of RVH. -Improved pulmonary function. -Improved cell survival. Increased number of small vessels. *Activation of PTEN/Akt pathway might be the underlying mechanism for these therapeutic effects*.
Willis et al., ^60^	Newborn term mice exposed to 75% O_2_ for 14 days	Human MSCs (umbilical cord, bone marrow)	dUC, TFF, DGC	NTA, TEM (Immu-nogold labelling), WB	Cell equivalent: 0.5 ×10^6^ MSCs cultured over 36 h.	Intravenous once at PND 4 •MSC-EVs •Myeloid cells pre-treated with MSC-EVs	Improved alveolarization, decreased fibrosis. Improved blood vessel count. -Decreased apoptosis. -Co-localization of EVs with myeloid cells in the lung tissue. A shift of myeloid cells towards an immune-suppressive phenotype was noted. *Therapeutic effects of EVs seem to be mediated by their ability to reprogram myeloid cells*.
Ai et al., ^65^	Newborn term rats exposed to 75% O_2_ for 14 days	Human MSCs (umbilical cord)	dUC	NTA, TEM, WB	Protein amount: 10 μg vs 15 μg of protein per dose of EV.	Intra-peritoneal once at PND 4	-Dose-dependent amelioration of alveolar simplification and fibrosis. -Improved vascular growth and pulmonary hypertension. -Delay in the trans-differentiation of AT2 cells into AT1 cells induced by hyperoxia. *This effect was suggested to be mediated by the downregulation of WNT5a*.
Abele et al., ^87^	Rats prenatally exposed to endotoxin at E20	Human MSCs (bone marrow)	Filtration DGC	Not specified. Reported to be in accord with 2018 MISEV guidelines	Cell equivalent: 0.25 ×10^6^ MSCs cultured over 36 h. NTA corresponding dose: 4.25 ×10^8^ particles.	Intra-amniotic at E20, along with endotoxin injection	-Reduction of placental inflammatory cytokines. -Improved postnatal distal lung growth and lung mechanics.
	E15 fetal rat lung explants exposed to endotoxin after 72 hours of in vitro culture	Human MSCs (bone marrow)	Filtration DGC	Not specified. Reported to be in accord with 2018 MISEV guidelines	Cell equivalent: 0.25 ×10^6^ MSCs cultured over 36 h. NTA corresponding dose: 4.25 ×10^8^ particles.	Added to the in vitro model at the same time with with the endotoxin	-Increased distal lung branching in the fetal lung explant. -Increased expression of VEGF and SPC.
Wu et al., ^66^	Newborn term mice exposed to 95% O_2_ for 3 days	Mouse MSCs (adipose-derived)	dUC	NTA, TEM, FC, WB	Protein amount: 30 ng vs 300 ng of protein per dose of EV.	Intra-tracheal once at PND 1	-Reduced lung wet-to-dry ratio, inflammation, oxidative stress, and apoptosis. -Upregulation of Nr2f2 expression (resulting from the upregulation of miR-21-5p expression) in lung tissue suggested as potential mediator of the therapeutic effects of EVs.
Lithopou-los et al., ^88^	Term mice exposed to endotoxin at PND 7 or 8, then to MV + 40% O2 at PND 9 or 10 for 4 hours	Human MSCs (umbilical cord)	dUC	SEM, NTA, DLS, WB	Protein amount: 0.005 μg of protein per gram of body weight. NTA corresponding dose: 1 ×10^6^ particles per gram of body weight.	Intra-tracheal once at PND 9 or 10 before ventilation	-Reduction in MLI. -Increased lung vessel density. -Increased expression of anti-inflammatory cytokines (IL-4, IL-10, IL-13). -No changes in the expression of pro-inflammatory cytokines (IL-1, IL-6, TNF-α). *These beneficial effects were not seen with EV-free conditioned media*.
Sharma et al., ^67^	Newborn term rats exposed to 85% O_2_ for 14 days	Human MSCs (umbilical cord vs bone marrow) [culture in bioreactor]	dUC	NTA	NTA: 3 doses evaluated: 2 ×10^8^, 12 ×10^8^ and 60 ×10^8^ particles per gram of body weight.	Intra-tracheal vs intravenous once at PND 3	•Both bone marrow- and umbilical cord-MSC EVs, in all 3 doses, and with both routes of administration: similar beneficial effects on lung regeneration and pulmonary hypertension. •Umbilical cord-MSC EVs: more promising effects on cardiac remodeling.
Bisaccia et al., ^72^	Newborn term rats exposed to 60% O_2_ for 14 days	Human MSCs (umbilical cord) [culture in bioreactor]	TFF	Cryo-TEM NTA	NTA: 6.4 ×10^9^ particles	Intra-tracheal at PNDs 3, 7, 10	-Reduced oxidative stress in both lungs and brain. -Improved pulmonary epithelial function. -Decreased lung fibrosis.
Saneh et al., ^58^	E17.5 fetal mice lung explants exposed in vitro to 95% O_2_ for 24 hours	hiPSCs and diPSCs (alveolar-like cells)	dUC UF SEC	TEM IFC NTA	NTA: 5 ×10^6^ particles per mL of media	Addition to media following hyperoxia exposure and for 48 h	-Improved parenchymal lung changes induced by hyperoxia. -Increased VEGF expression in lung tissue. -Increased antioxidants expression in lung tissue.
Albertine et al., ^86^	Preterm lambs at 129 days, exposed to mechanical ventilation for 6 to 7 days	Human MSCs (bone marrow)	dUC, TFF, DGC	NTA, TEM (Immu-nogold labelling), WB	NTA: 2 ×10^11^ particles	Intravenous at hour of life 6 and at hour of life 72	-Decreased respiratory severity score, oxygenation index, alveolar-to-arterial oxygen gradient, septal wall thickness. -Increased radial alveolar count and alveolar capillary density. -More abundant VEGF receptor 2. -Better tolerance of enteral feeds. -Improved growth trend.

*Akt* alpha serine/threonine-protein kinase, also known as PKB (protein kinase B), *AT1* alveolar type I, *AT2* alveolar type II, *DGC* density gradient centrifugation, *diPSC* human induced pluripotent stem cells (hiPSCs) differentiated into alveolar-lung cell, *DLS* dynamic light scattering, *dUC* differential (or sequential) ultracentrifugation, from low- to high-speed, *E* embryonic day, *ELISA* enzyme-linked immunosorbent assay, *EV* extracellular vesicle, *FC* flow cytometry, *hiPSC* human induced pluripotent stem cell, *IFC* imaging flow cytometry, *IL* interleukin, *LSC* low-speed centrifugation, *miR* microRNA, *MISEV* Minimal Information for Studies of Extracellular Vesicles 2018, *MLI* mean linear intercept, *MSC* mesenchymal stem cell, *MV* mechanical ventilation, *Nrf2f2* nuclear receptor subfamily 2, group F, member 2, *NTA* nanoparticle tracking analysis, *O2* oxygen, *PND* postnatal day, *PTEN* phosphatase and tensin homolog, *RVH* right ventricular hypertrophy, *SEC* size-exclusion chromatography, *SEM* scanning electron microscopy, *SPC* surfactant protein C, *TEM* transmission electron microscopy, *TNF* tumor necrosis factor, *TTF* tangential flow filtration (also known as cross-flow filtration, as opposed to the term “filtration” used to refer to the traditional dead-end filtration where fluid flows perpendicular to the membrane), *UF* ultrafiltration, *VEGF* vascular endothelial growth factor, *WB* western blot, *Wnt5a* transduction signal created from the names Wingless and Int-1, family member 5a.

Despite the significant variability among studies in terms of animal model, stem cell source, EV isolation technique, route and timing of administration, dosages, and goal outcomes, MSC-EVs seem to be promising in repairing parenchymal and vascular injury in the neonatal lung. Willis et al. were the first to show these therapeutic benefits,^59^ using a rigorous and structured approach for the isolation and characterization of EVs. The same group continued to publish their findings over the past years, further elucidating potential pathways that could explain the effects of MSC-EVs on the injured neonatal lung. Immunomodulation of the macrophage phenotypes M1 (proinflammatory) / M2 (anti-inflammatory) in the lung is one of the proposed mechanisms, as evidenced by the ability of MSC-EVs to suppress some of the markers associated with polarized macrophages in vitro and in vivo.^59^ When tracked, these EVs seemed to co-localize and directly interact with myeloid cells within the host lung, promoting a CCR2-negative myeloid phenotype.^60^ CCR2 is the receptor for the chemokine ligand CCL2, and the activation of the CCR2-CCL2 axis is critical for the chemotactic response to occur. These findings suggest that the therapeutic effects of MSC-EVs may be in part mediated by their ability to induce a shift of pulmonary myeloid cells towards an immunosuppressive phenotype.^60^

Additional mechanisms and pathways were also suggested by other groups, including: VEGF transfer from MSC-EVs to the host lung^61,62^; detection inside MSC-EVs of the immunosuppressive tumor necrosis factor-stimulated gene-6 TSG-6, known to promote macrophage shift toward the anti-inflammatory phenotype M2;^63^ activation of the signaling pathway PTEN/Akt, known to regulate multiple biological processes including cell proliferation, growth and apoptosis;^64^ delay of the hyperoxia-induced trans-differentiation of alveolar cells AT2 to AT1, mediated by the downregulation of the transduction signal Wnt5a;^65^ transfer of the microRNA miR-21-5p into the host lung, resulting in increased expression of nuclear receptor Nr2f2, which in turn interacts with the enhancer binding protein C/EBPα to promote lung repair.^66^ Overall, these studies indicate that MSC-EVs exhibit an exciting therapeutic potential to treat premature lung disease and warrant further investigation for clinically translatable applications.

Towards this direction, a multicenter randomized controlled trial (NCT03857841) was launched in 2019 to evaluate the safety and tolerability of MSC-derived EVs (UNEX-42) in high-risk preterm infants born before 27-weeks’ gestation and with a birth weight of 750 grams or less. The study was meant to include intubated infants 3-14 days old, not expected to be extubated in the 24 h following their enrollment, and who meet the following criteria: any oxygen requirement for those born before 25-weeks’ gestation, or inspired O2 fraction of at least 35% for those born between 25- and 27-weeks’ gestation. The study was however discontinued by the sponsor in 2021 due to a business decision, reportedly not related to reasons of safety or efficacy. Therefore, the potential of stem cell derived-EVs for clinical translation remains to be explored.

## Stem cell-derived EVs for neonatal lung injury: Heterogeneity and limitations of the available studies

While the available preclinical trials show a promising role for stem cell-derived EVs in repairing neonatal lung injury, significant heterogeneity exists among studies and many questions remain unanswered in regard to their effectiveness and overall safety. Standardization and establishment of rigorous and clear protocols are needed for future clinically translatable applications.

### Limitations regarding stem cell source and culture conditions

Research involving EVs in neonatal lung diseases mostly used MSCs as cell sources, given their well-known anti-inflammatory, anti-oxidant, immunomodulator, and regenerative effects. However, these MSCs were derived from different donors, at different ages, and from different tissues (umbilical cord, bone marrow, or adipose tissue). Chaubey interestingly noted that umbilical cord MSCs obtained from preterm donors born at 25-weeks’ gestation generated EVs that were more effective in repairing neonatal lung injury than those collected from donors born at 30-weeks’ gestation.^63^ Willis et al. and Sharma et al. compared the efficacy of allogeneic umbilical cord MSC-EVs and bone marrow MSC-EVs in repairing neonatal lung injury using the same experimental design, and showed similar promising results with no significant differences between the two treatments.^59,67^

To the best of our knowledge, only two studies investigated EVs derived from hiPSCs and their alveolar progenies, one of them was limited by the EV isolation technique^21^ and the other by the in vitro nature of the experiment.^58^ HiPSC-EVs would be interesting to further investigate in BPD, especially that some proteomic analysis have shown that they carry fibroblast growth factor-2 (FGF2), vascular endothelial growth factor (VEGF), anti-inflammatory interleukin-4 (IL-4), and proteins involved in epidermal growth factor receptor (EGFR) interactions, more abundantly than MSC-EVs.^68^ Analysis performed by our group also showed that hiPSC-EVs were enriched in proteins implicated in the processes of alveolarization and angiogenesis, notably those involved in hypoxia-inducible factor 1 alpha (HIF1α), VEGF and endothelial NO synthase (eNOS) activation pathways.^58^

In regard to cell culture conditions, it is now known that the profile of vesicles released by a specific cell type is directly influenced by environmental growth conditions, such as the culture system, cell seeding density and passage, media composition, and oxygen tension.^69^ When these conditions are variable among studies, it becomes difficult to compare the results, even if EV cell source is the same. Furthermore, cell culture conditions were described in only a few of the EV-based BPD studies listed in Table 3.

Porzionato,^70,71^ Sharma^67^ and Bisaccia^72^ used bioreactors to expand their MSCs before the collection of EVs, while other investigators used conventional dishes or flasks. The biomimetic properties of extracellular matrix scaffolds allow for more physiologic cell adhesion, survival, interaction, and proliferation, resulting in improved EV generation. It has been shown that three-dimensional (3D) culture systems (such as bioreactors) result in a much larger EV yield than traditional two-dimensional (2D) systems (dishes or flasks). The increased production can be up to 40-fold of EVs per mL of conditioned media.^73^

Cell density may also impact EV production and release. Seeding MSCs at low densities has been shown to result in a more rapid cellular proliferation and a larger EV production. When contact signaling is reduced, MSCs seem to compensate by increasing EV generation for paracrine intercellular communication.^74,75^ Conversely, higher cell seeding densities may lead to contact inhibition and/or cellular death, resulting in decreased EV production.^69^ Therefore, quantification of EVs in terms of cell equivalents, though widely used in EV studies listed in Table 3, may be misleading, since cell density seems to be a more important determinant of EV yield than absolute cell numbers. A higher cell number doesn’t necessarily result in a larger EV quantity, especially when seeding is performed at high densities that reduce the need for contactless communication.

Increased cell passage, especially beyond passage 4, may reduce EV bioactivity. With cell aging, chromosomal telomeres progressively shorten. The resulting “telomere uncapping” induces an increased transcription of telomere repeat-containing RNAs, also known as TERRA. These non-coding RNAs are enriched in EVs collected from senescent cells and are known to stimulate the production of inflammatory cytokines.^76,77^ The profile of EVs derived from the same stem cell can thus shift from a protective phenotype when generated at low passages to a pro-inflammatory phenotype when collected from higher cell passages. If cell passage is not consistent among EV batches used for the same experiment, results become difficult to interpret and to generalize.

Media content is another major determinant of EVs profile. Xeno-free serum (deprived of animal-derived components) enhances EV production by human MSCs. These EVs were found to be more angiogenic and less immunogenic than those obtained when fetal bovine serum (FBS) was added to the medium.^78^ Priming cells by adding soluble growth factors to the media may also impact EVs characteristics and bioactivity.^79^ For instance, platelet-derived growth factor (PDGF) was shown to stimulate adipose tissue-derived MSCs to produce EVs with a higher angiogenic potential.^80^ It is important here to recognize that a specific EV population may be beneficial for one disease process, but not for another one.^52^ Pre-conditioning stem cells to generate EVs with pro-angiogenic, anti-inflammatory and anti-oxidant potential, may enhance their therapeutic effects in BPD models.

Regarding oxygen tension used for stem cell culture in EV-based BPD studies, it was either reported as atmospheric (21%) or unspecified. It would be interesting to study the profile of EVs derived from stem cells cultured under supra-atmospheric oxygen tensions. Would the cells respond by releasing vesicles with an enhanced cytoprotective and anti-oxidant potential? Or would their profile divert to a pro-inflammatory phenotype, similarly to what happens with the endogenous pulmonary pool of MSCs when exposed to inflammation? Further studies are needed to explore the bioactive potential of EVs derived from stem cells under non-physiologic oxygen tensions and their effects in animal models of BPD.

### Heterogeneity in EV isolation and characterization techniques

**Fig. 3:**
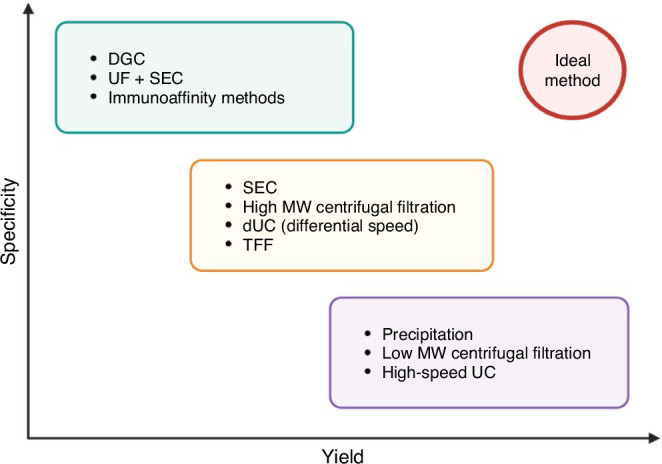
Common EV isolation techniques, categorized according to their yield and specificity. The ideal goal of obtaining a purified EV population with both high yield and high specificity remains unrealistic with the currently available techniques. DGC density gradient centrifugation, dUC differential or sequential ultracentrifugation (low-to-high speed), MW molecular weight, SEC size-exclusion chromatography, UC ultracentrifugation, UF ultrafiltration, TTF tangential flow filtration (also known as cross-flow filtration, as opposed to the term “filtration” used to refer to the traditional dead-end filtration where fluid flows perpendicular to the membrane). [Created with BioRender].

Multiple techniques have been developed to isolate EVs from biological samples and/or conditioned media. The most commonly used methods are categorized in Fig. 3, based on their yield and purity.^51^ Comparative reports have shown significant heterogeneity among these techniques in terms of EV concentration, particle size distribution, surface markers and proteomic profile.^81,82^ Different isolation techniques may then result in EVs with different bioactive potentials. It is thus difficult to combine the results of the available EV-based BPD studies (Table 3) since they were not performed with comparable methods for EV isolation. Additionally, while ultracentrifugation was widely used to collect EVs, spinning speeds were variable among studies. Therefore, the outcomes of each study reflect the bioactive potential of the EV population used, and results cannot be generalized to all the EV populations recovered from a certain stem cell type.

It is then important to characterize the isolated EV samples and to report which population was investigated. Many EV-based studies in neonatal lung injury did not fulfill all the characterization criteria recommended by MISEV 2018 (Table 4). The requirements generally missed included the identification of at least one cytosolic protein, the assessment for the presence of contaminants, and the topology specification for components believed to be associated with a certain function.^83^ Following standardized characterization guidelines in future studies may help identify the EV populations that are most effective in repairing injury in the preterm lung.

**Table 4:** Recommendations of MISEV 2018 for EV characterization.

Characterization of EVs as per MISEV 2018 recommendations	
Quantitative description of both the source of EVs and the EV preparation	Source of EVs: number of cultured cells, starting volume of media or biofluid, …. EV preparation: total particle number, total protein amount, total lipid content, ….
Protein composition	At least 3 positive protein markers (including both transmembrane and cytosolic). At least one negative protein marker. Testing for non-vesicular co-isolated contaminants.
Single vesicle analysis, with at least two different techniques	Imaging and estimation of biophysical features, such as size, morphology, and scattering or fluorescent properties.
Topology of EV components (luminal vs surface)	Especially in studies aiming to associate a component to specific function.

Adapted from: Théry C, et al. Minimal information for studies of extracellular vesicles 2018 (MISEV2018): a position statement of the International Society for Extracellular Vesicles and update of the MISEV2014 guidelines. Journal of Extracellular Vesicles. 2018;7(1):1535750.

Furthermore, most of the preclinical EV-based studies for neonatal lung injury did not specify whether fresh or stored EVs were used in the experiments, and whether EV characterization was performed before storage or directly before use. Storage conditions significantly affect EV concentration and stability. Recovery and purity both decrease over time.^84^ EV concentration was reduced by around 60% within only 2 weeks of storage in Phosphate Buffered Saline (PBS) at -20 degrees Celsius. A lower storage temperature of -80 degrees Celsius seemed to preserve the samples for a relatively longer period. Upon testing different buffers, Görgens et al. noted that supplementing PBS with human albumin and trehalose significantly improved not only the short- and long-term preservation of EVs (up to 2 years), but also their stability throughout multiple freeze-thaw cycles.^85^

### Heterogeneity in the preterm lung model and the experimental design

Normal lung development occurs in 5 stages: embryonic, pseudo-glandular, canalicular, saccular, and alveolar. Infants born at term are already at the alveolar stage of their lung development. In contrast, term rodents are born during the mid- to late- saccular stage of lung development (embryonic day 21-22), equivalent to a gestational age of 30- to 36- weeks in humans, thus their widespread use in BPD studies.^5^ Most of the EV-based BPD studies listed in Table 3 used term rodents to mimic BPD. Recently, Albertine et al. applied EV therapeutics to a larger animal model by using preterm lambs born at around 129 days’ gestation, equivalent to 28-weeks in human gestation and corresponding to the early saccular stage of lung development.^86^ This larger animal model is highly promising, as it was carefully designed to mimic the conditions that very preterm infants are exposed to at birth and the interventions they receive, including Surfactant and Caffeine administration. Nonetheless, models that mimic extreme preterm birth when the lungs are still at the canalicular stage of their development are still lacking. Severe BPD is nowadays mostly affecting infants born at the canalicular stage. Therefore, it’s important to develop models that mimic the arrest of development early on, either through extreme preterm birth, or through antenatal injuries to the developing lung similarly to the model used by Abele et al.^87^

In terms of exposures, most of the EV-based BPD studies used high oxygen levels to induce lung injury. Oxygen concentrations and duration of exposure were significantly variable across studies. Abele et al. used endotoxin to trigger inflammation and induce injury in fetal rat lungs at E15 in vitro and E20 in vivo.^87^ This is a promising BPD model since it recapitulates injury at the canalicular stage. Another interesting model by Lithopoulos et al. was designed using endotoxin exposure along with hyperoxia and mechanical ventilation to mimic BPD changes.^88^ This combination of exposures is a good representation of the multitude of risk factors that contribute to disease development in preterm infants. Developing preclinical models that better recapitulate the hallmark symptoms, functional and histological findings of BPD will enable the accelerated development of EV-based therapies.

The timing of EV administration (preventively or therapeutically), the route of administration (intravenous, intra-oral, intra-tracheal, or intraperitoneal), and EV dosage were also significantly variable among studies. Furthermore, lungs were assessed at different timepoints and using different outcome measures. This heterogeneity makes it hard to interpret the results. Li et al. found that proinflammatory markers (IL-6 and TNF-α) were reduced in the bronchoalveolar fluid of BPD models following MSC-EV administration,^89^ while Lithopoulos et al. did not note any changes in the expression of these markers.^88^ However, the two experiments were performed using different lung injury models, different cell sources (amnion-derived vs. umbilical cord MSCs), different speeds of centrifugation to isolate EVs, different doses (absolute dose vs. weight-based dose measured in protein amount), different timing of EV administration relatively to the exposure, and different timepoints for outcome assessment. Thus, no reliable conclusions can be drawn from these studies. EV-based BPD experiments still need to be optimized to answer the questions of when, how, how much, and how many times stem cell-derived EVs should be administered to achieve the highest efficacy without causing adverse effects.

## Stem cell-derived EVs for neonatal lung injury: challenges and future directions

EV-based therapies are solely experimental at this stage. Multiple challenges need to be addressed to ease the transition towards clinical translation (Table 5).

**Table 5:** Challenges associated with the use of stem cell-derived EVs as a therapeutic for BPD.

Challenges that need to be addressed to facilitate the clinical application of stem cell-derived EVs in preterm lung disease
1. Optimizing the experimental preterm lung model. The lung model should ideally mimic the “new BPD” lung in regard to its pathogenesis and histologic hallmarks. 2. Finding the optimal stem cell source. Large-scale production of effective EVs is ultimately necessary for any clinical application. 3. Optimizing cell culture environment: • Culture system (preferably 3D). • Seeding density and cell passage. • Media composition. • Oxygen tension. 4. Standardizing the technique used for EV isolation. *Ensuring reproducibility and replicability of the technique used.* 5. Standardizing EV characterization as per MISEV guidelines. 6. Optimizing storage conditions that preserve EV bioactivity. Clinical application will ultimately require the availability of vesicles “off-the-shelf” and “ready-to-use”. 7. Developing a standardized experimental design for EV treatment: • EV dosage and number of doses required. • Timing and route of administration. • Timing of lung tissue assessment. • Methods used for lung tissue assessment.
8. Understanding the pharmacokinetics of EVs. 9. Establishing EV safety profile.

There is first a need for better experimental models able to mimic preterm lung tissue at-risk for BPD (in vitro models such as bioprinted organotypic constructs or in vivo animal models at the late canalicular or early saccular stage of their lung development). Insults need to be inflicted to the extreme preterm lung and need to reflect the pathogenesis of the “new” disease using, for example, a multitude of pre- and post-natal exposures, such as endotoxin, nutritional deficits, mechanical ventilation, oxygen toxicity, … among others.

Second, we need to find the optimal stem cell source and culture conditions that allow for large scale-production of functional and effective EVs. While MSC-EVs seem to be highly effective in preclinical studies, the role of hiPSC-EVs is yet to be determined. Preconditioning cells to generate EVs with pro-angiogenic, anti-inflammatory and antioxidant potential will further enhance efficacy. Genetic reprogramming of the cell source is also another research area that may help bio-engineer vesicles with a very specific bioactive profile. For instance, transfecting bone marrow-derived MSCs with miR-34c-5p resulted in the production of EVs that were capable of improving parenchymal changes and attenuating inflammation in a mouse BPD model.^90^ More research is needed to improve our understanding of the genetic and transcriptomic mechanisms underlying the pathogenesis of BPD. Once the beneficial or protective genes, mRNAs or miRNAs are identified, the efficacy of stem cell-derived EVs can be enhanced by genetically modifying or reprogramming the cell source. Bioengineered EVs can thus be used as a vehicle for gene therapies that could, in the future, be designed to target specific BPD phenotypes.

Additionally, EV isolation and characterization protocols need to be standardized to ensure reproducibility and replicability of experimental studies. While high-speed ultracentrifugation has been widely used in the past to collect EVs, this technique alone results in a heterogeneous sample where non-vesicular contaminants are largely present. Combining multiple isolation techniques (such as dUC with DGC, or UF with SEC) increases specificity and results in higher homogeneity of the EV population. Developing clinical-grade good manufacturing practice EV products that are homogeneous and reproducible is key to bringing these technologies closer to clinical applications. On another hand, while fresh samples can be an option for preclinical BPD studies, clinical translation will ultimately require the availability of “ready-to-use” particles. Standardized and efficient storage protocols are thus needed.

It’s also important to understand the pharmacokinetics of EVs which will ultimately help determine the required dose and frequency needed to achieve the desired effect. Biodistribution largely depends on the route of administration. It has been shown that, following intravenous injection into animal models, large EVs localize in the lung tissue within the first hour, while small EVs are detected in the lungs mostly between 2 and 12 h after their administration. Levels decreased afterwards.^91^ Is this short period of time enough for EVs to be effective and deliver their cargo to the recipient pulmonary cells? Will a single dose be enough for a chronic disease such as BPD? MSCs delivered intra-tracheally to BPD models resulted in transient engraftment, but efficacy was still detected several weeks following treatment.^6^ EVs are likely to remain in the lung tissue for a lesser period of time. Will the same long-term efficacy be obtained, or are additional doses needed? Further studies are needed to address these concerns and enhance the therapeutic efficacy of these regenerative therapies for BPD.

Lastly, safety studies are still lacking. Establishing a safety profile requires a thorough evaluation for potential inherent toxicities and the development of manufacturing regulations with robust quality control criteria.^92^ Implementing a safe precision dosing approach, preferably weight-based, is important for neonatal applications. Developing and validating in vitro potency assays is essential to pre-test EV preparations intended to be used in therapeutics. In summary, to accelerate clinical applications, a clear framework that defines quality and safety needs to be established. The collaboration of stakeholders, including manufacturers, researchers, clinicians, and regulatory authorities, helps implement robust compliance programs when designing EV manufacturing facilities and developing good manufacturing practice products, which will ultimately facilitate the transition towards clinical trials.

## Conclusion

In conclusion, experimental BPD studies have shown, despite their heterogeneity, a promising role of stem cell-derived EVs in lung tissue regeneration and repair. Transitioning towards clinical translation still requires standardization of EV isolation and characterization protocols, establishment of pharmacokinetic and safety profiles, and development of a robust regulatory framework for manufacturing purposes. A better understanding of the mechanism of action of stem cell-derived EVs in the preterm lung is also needed. Researchers may then enhance the therapeutic potential of these nano-packages by preconditioning or genetically reprogramming the parent cells to generate a specific EV population capable of preventing and/or repairing the parenchymal and vascular lung disease associated with prematurity. Identifying the soluble factors or nucleic acids that mediate the therapeutic effects of EVs may also pave the way for the bio-engineering of potent vesicles specifically designed for functional applications in neonatal lung diseases.
